# Pharma 4.0: A deep dive top management commitment to successful Lean 4.0 implementation in Ghanaian pharma manufacturing sector

**DOI:** 10.1016/j.heliyon.2024.e36677

**Published:** 2024-08-29

**Authors:** Michelle Grace Tetteh, Sumit Gupta, Mukesh Kumar, Hana Trollman, Konstantinos Salonitis, Sandeep Jagtap

**Affiliations:** a Sustainable Manufacturing Systems Centre, Cranfield University, Cranfield, MK43 0AL, UK; b Department of Mechanical Engineering, A.S.E.T., Amity University, Uttar Pradesh, Noida, 201313, India; c National Institute of Technology Patna, Patna, 800005, India; d School of Business, University of Leicester, Leicester, LE2 1RQ, UK; e Department of Industrial Management and Logistics, Faculty of Engineering, Lund University, Lund, Sweden

**Keywords:** Lean manufacturing, Industry 4.0, Lean 4.0, Corporate performance, Pharmaceuticals, Pharma 4.0, Top management

## Abstract

The primary aim of this study is to assess the significance of top management commitment in the context of Lean 4.0 implementation within the pharmaceutical manufacturing industry in Ghana. The study seeks to understand and evaluate the overall effectiveness and achievements associated with adopting Lean 4.0. Employing a positivist mindset, the research utilizes an explanatory quantitative research design and a survey technique. Data collected from 181 employees of pharmaceutical companies in Ghana undergo analysis using SmartPLS (version 4) and IBM SPSS version 26. The study employs a combination of descriptive statistics to summarise data characteristics and inferential statistics to test various hypotheses related to Lean 4.0 adoption. The analysis reveals that the successful integration of lean methods and Industry 4.0 technologies requires meticulous management. Simultaneously, individual implementations of lean principles and Industry 4.0 technologies positively impact business performance. Surprisingly, the study does not observe a substantial positive influence of Lean 4.0 on corporate performance, suggesting that immediate improvements in efficiency or profitability may not result from the adoption of this framework. This research contributes to the field by highlighting the need for careful management in integrating lean methods and Industry 4.0 technologies. It also emphasizes the positive impacts of lean principles and Industry 4.0 technology on business performance. The unexpected finding regarding the lack of immediate improvements in corporate efficiency or profitability with Lean 4.0 adoption prompts considerations of initial implementation challenges or the organization's need for time to adapt to this integrated approach.

## 1 Introduction

The Ghanaian pharmaceutical industry plays a pivotal role in the nation's healthcare system by ensuring the timely and efficient delivery of essential medicines and therapeutics to the population. Comprising 30 % domestically manufactured medications and 70 % imported medicines, largely from India and China [1,2], the industry reached a projected value of USD 250 million at the retail price level by 2005, with an expected growth rate of 6–8% [2]. To eliminate waste, improve quality, and increase efficiency in the production and delivery of goods and services. However, using digital Industry 4.0 technologies, such as the Internet of Things, cloud computing, big data analytics, artificial intelligence, and robotics in manufacturing is required. Ghana pharmaceutical companies may require Lean 4.0 practices implementation. Lean 4.0 is the combination of Lean manufacturing and Industry 4.0. Pharmaceutical organisations necessitate the incorporation of analytical methodologies, such as simulation, data analytics, and optimisation, to get a more comprehensive comprehension of the data produced. Nevertheless, the essential technologies are predominantly prototypes that have not yet been widely implemented for commercial use. The widespread implementation of Industry 4.0 will not progress unless the associated technology becomes economically viable.

Despite boasting a highly efficient supply and distribution network that extends the availability of effective medicines to West Africa and parts of sub-Saharan Africa, the local pharmaceutical industry faces challenges in enhancing its manufacturing capacity [3,4]. While Ghana presently serves as a hub for pharmaceutical production and delivery to the nearly 300 million residents within the Economic Community of West African States (ECOWAS) [2,5], only 900 medications are currently manufactured domestically [5].

As the industry evolves, prioritizing the adoption of Lean principles and Industry 4.0 technologies becomes imperative for enhancing efficiency and quality in pharmaceutical production [4,6]. The integration of Lean 4.0, an amalgamation of Industry 4.0 and Lean principles into pharmaceutical manufacturing, holds transformative potential by seamlessly incorporating technologies such as the Internet of Things, Blockchain, Artificial Intelligence, etc. However, challenges persist in management involvement in implementing Lean 4.0 for the quality manufacture of medications, necessitating effective resolution [7,8]. Previous successful implementations of Lean concepts in pharmaceutical facilities have resulted in significant improvements in efficiency, quality, and cost reduction [9]. These achievements are expected to inspire the sector to reformulate its business strategies and embrace Lean and Pharma 4.0 methodologies [10]. For the Ghanaian pharmaceutical industry to successfully adopt Lean 4.0, firm commitment from senior management is crucial. Currently, the sector stands at a critical juncture, embracing the revolutionary capabilities of Lean manufacturing and Industry 4.0 technologies, proven to enhance efficiency and quality [6,11,12].

The transformation of the pharmaceutical business in Ghana has been significant over time, originating during the colonial period with the primary goal of importing drugs to meet local needs [13,14]. This research aims to underscore the vital significance of top management dedication in effectively implementing Lean 4.0 in Ghana's pharmaceutical manufacturing, exploring the pivotal role of senior management commitment in promoting the adoption of Lean 4.0 concepts. Through this study following research questions are answered from the perspective of the Ghanaian pharmaceutical sector.

• How does top management commitment influence the successful implementation of Lean principles in the Ghanaian pharmaceutical sector?
• What is the impact of top management commitment on the adoption and successful implementation of Industry 4.0 in the Ghanaian pharmaceutical sector?
• How do Lean principles and Industry 4.0 interact to influence the adoption and successful implementation of Lean 4.0 in the Ghanaian pharmaceutical sector?
• What is the relationship between Lean 4.0 implementation and corporate performance in the Ghanaian pharmaceutical manufacturing sector?

### 1.1 Research objectives

The primary aim of this research is to identify the impact of top management implementing Lean 4.0 principles in the pharmaceutical manufacturing industry in Ghana. More precisely, the project aims to.

• Investigate the relationship between Top Management Commitment and Lean.
• Investigate the relationship between Top Management Commitment and Industry 4.0.
• Evaluate the combined impact of Lean and Industry 4.0.
• Investigate the relationship between Lean 4.0 and corporate performance.

Thus, towards fulfilling the research objective a comprehensive survey on the Ghanaian pharmaceutical sector has been performed to identify the impact of top management implementing Lean 4.0 principles in the pharmaceutical manufacturing industry in Ghana. According to Upper Echelons Theory (UET), the values, cognitive styles, and experiences of senior managers have an impact on their ability to make strategic decisions and the success of their organisations. By analyzing the roles that leadership, vision, culture, and innovation play in accelerating digital transformation and generating competitive advantage, UET may assist in understanding how the pharmaceutical industry might implement Lean 4.0. RBT contends that a company's success and sustainability are determined by its resources and capabilities, which include its assets, knowledge, skills, and procedures. By examining how the application of digital technologies may improve the effectiveness, quality, adaptability, and resilience of the pharmaceutical supply chain as well as the value generation and capture for the stakeholders, Resource-based theory (RBT) can assist in understanding how the pharma sector can benefit from Lean 4.0. A set of questionnaires has been prepared and responses from the Ghanaian pharmaceutical sector have been collected. The responses were used to test a research model prepared based on UET and RBT. The research model has been tested on a partial least square-based structural equation modelling technique. The unique findings of this research explore the top management role in the implementation of Lean 4.0 in the pharma industry which contributes to both theory and practice.

The introductory section serves as the Introduction, providing a preliminary synopsis of the findings. It delves into the historical and contextual aspects of the topic, thereby establishing the framework for the inquiry. This section also furnishes the background, research questions, and objectives, offering a clear indication of the research's intentions within the pharmaceutical industry of Ghana. The second section immerses into a thorough Literature Review, enhancing the overall comprehension of the subject matter. It commences with a review of methodology and constructs that consider the conceptual overview in the context of the research, encompassing Lean 4.0 and Top Management Commitment. This section also addresses the research gap and further refines the hypotheses accordingly. The third section, Methodology, details the survey questionnaire's development. Moreover, it outlines the methodologies for data collection, the analytical procedures utilized for interpreting the acquired data, and the statistical tools used. The fourth section, titled "Data Analyses,” comprehensively presents the study's findings. The section concludes by examining the results and establishing their connection to the study questions and goals stated in the first section. The fifth section, titled "Discussions,” provides a concise overview of the study's results, connecting these results to the research goals. The sixth section offers research implications that consider both managerial and theoretical perspectives arising from the results, presenting a conclusive amalgamation of the study. Subsequently, practical and implementable suggestions are provided, derived from the aforementioned findings. The seventh section concludes by offering recommendations for future study, highlighting prospective domains that may benefit from further scholarly investigation.

## 2 Literature review

### 2.1 Lean manufacturing

Lean techniques serve as the foundation for enhancing operational efficiency and fostering continuous improvement across various sectors. Rooted in the principles of the Toyota Production System, these techniques are designed to eliminate waste and streamline operations, ultimately maximizing customer value. In the realm of pharma manufacturing, the implementation of lean manufacturing practices can effectively reduce waste without compromising quality or productivity [[15], [16], [17]].

Achieving success in the adoption of lean techniques requires businesses to possess a robust understanding of the distinctions between conventional manufacturing and lean manufacturing practices [15,16,18,19]. This foundational knowledge is essential for navigating the nuances of lean methodologies and ensuring their seamless integration into existing manufacturing processes. Notwithstanding all of Lean's advantages and applications, experts think the methodology is out of date and won't be able to stay up with contemporary client expectations and trends. According to Dennis and Detlef (2015), while the Lean methodology has been incredibly successful, its capacity to produce highly customized goods is limited. The industrial automation idea known as "industry 4.0″ has the potential to meet these expanding demands.

### 2.2 Industry 4.0

The literature presents various definitions of Industry 4.0, highlighting its multifaceted nature. This concept encapsulates diverse elements that enable automated processes without human intervention [[20], [21], [22]]. Industry 4.0 has a big influence on the industrial environment and changes a lot of things about how jobs are done, according to Adam et al., 2016. The fourth industrial revolution is defined as the use of intelligent technologies and systems that allow for information collecting and online interaction between products, operations, distributors, and purchasers (Sven et al. [2018]). The goal of industry 4.0, according to Keliang 2014], is to provide a dynamic manufacturing system for customized and electronic goods and services that permits direct communication between the products and services during the manufacturing process. Embracing cyber-physical systems (CPS), the Internet of Things (IoT), the Industrial Internet of Things (IIoT), Big Data Analytics, Artificial Intelligence (AI), Machine Learning (ML), Cloud Computing, Additive Manufacturing (3D Printing), Advanced Robotics, Augmented Reality (AR), and Virtual Reality (VR) [23]. Industry 4.0 represents a transformative movement towards automation and data interchange within manufacturing technologies and processes [24].

### 2.3 Lean 4.0

Lean 4.0 signifies the fusion of Lean principles with Industry 4.0 technology, harmonizing the conceptual and process-oriented methodologies of Lean with the technology-centric tools of Industry 4.0. This integration is pursued with the overarching goal of achieving operational excellence [25,26]. The synergy between these two influential theories lays the groundwork for a transformative era in organizational effectiveness and productivity [27]. Osti (2020) asserts that the principles of value creation in Industry 4.0, such as operational efficiency, reduced production costs, and quality assurance, closely align with those of Lean management, which prioritises minimising internal waste and enhancing customer satisfaction. Both approaches ensure ongoing improvement and the delivery of products that meet customer expectations. Christian (2012) emphasized that a crucial element of the Industry 4.0 landscape and technology is the efficient utilization of information and communication technology (ICT). Osti (2020) states that lean manufacturing is a valuable asset in effectively utilizing industry 4.0 technologies. Nai et al. (2019) observed that Industry 4.0 technology has a positive impact on several types of waste generated during production using lean manufacturing. Stephen and Brian (2020) propose that digital lean utilizes industry 4.0 tools to provide operators with precise and comprehensive information, facilitating the identification and elimination of waste. This technique enables faster waste detection and mitigation compared to traditional lean methods.

The emergence of Lean 4.0 is a tangible manifestation of the ongoing commitment to refining organizational procedures and achieving heightened effectiveness. As Lean concepts evolved and technology advanced, the synergistic potential between these domains became increasingly evident. The Lean 4.0 methodology strives to leverage technology's capabilities to automate processes, provide critical information, and expedite operations—all while upholding the fundamental principles of Lean ([14,28,29].

Table 1 reveals research in academia has focused on the investigation of Industry 4.0 technology and lean manufacturing ideas, particularly in the manufacturing sectors. Multiple research studies have explored the impact of new technologies such as artificial intelligence, the Internet of Things (IoT), and big data analytics on conventional manufacturing methods. These technologies are being used to improve efficiency, productivity, and sustainability in manufacturing. The improvements known as Industry 4.0 signify a fundamental change in manufacturing approaches, focusing on automation, connectivity, and data-driven decision-making [30]. Although several papers have explored the interaction between Industry 4.0 technology and lean manufacturing concepts with top management commitment, the majority of these studies have been carried out in industrialised nations. Nevertheless, there is an increasing acknowledgement of the need to broaden the scope of this study to include more sectors and areas, namely rising economies and specialized businesses such as the food industry. The food business, known for its intricate supply chains, rigorous quality requirements, and sustainability issues, offers distinct possibilities and difficulties in incorporating business 4.0 technology and lean manufacturing concepts [31].

**Table 1:** Recent Article in manufacturing sectors with Lean and Industry 4.0

Author & year	Article	Industry	Outcomes
[32]	“Understanding the adoption of Industry 4.0 technologies in improving environmental sustainability”	Manufacturing	Insights into how Industry 4.0 can drive sustainable manufacturing practices by leveraging advanced technologies and optimising production processes.
[33]	“The fourth industrial revolution in the food industry—Part I: Industry 4.0 technologies”	Food industry,	are centred on either manufacturing in a general context or highlighting the role of Industry 4.0 technologies in addressing these challenges and driving sustainable development in the food sector.
[20]	“Industry 4.0 technologies: implementation patterns in manufacturing companies”	Manufacturing	propose a conceptual framework for Industry 4.0 technologies, dividing them into front-end and base technologies. Front-end technologies include Smart Manufacturing, Smart Products, Smart Supply Chain, and Smart Working dimensions, while base technologies comprise the Internet of Things, cloud services, big data, and analytics
[34]	“Lean manufacturing techniques and its implementation: A review”	Manufacturing	to extract approaches for improving the implementation of Lean manufacturing principles to enhance productivity while simultaneously reducing product costs. It aims to shed light on Lean waste reduction methods and provide insights into the current state of Lean manufacturing.
[35]	“Study and implementation of lean manufacturing strategies: A literature review”	Manufacturing	a critical review of lean manufacturing and reverse engineering for joint propeller shafts, categorizing them systematically based on definitions, design bases, organizational bases, material bases, and tool use bases. Additionally, the article analyzes lean manufacturing concepts, waste reduction strategies, implementation obstacles, and performance measurement tools.
[36]	“Literature Review of the Benefits of Lean Manufacturing on Industrial Performance and Proposed Applications in the Defence Industries”	Defence Industry	to provide insights into the benefits of implementing Lean Manufacturing across different industries and to offer recommendations tailored to the defence industry.
[37]	“The effect of the digital supply chain on lean manufacturing: A structural equation modelling approach”	Electronic Industry	provide insights into the relationship between digital supply chain dimensions and lean manufacturing and offer guidance for organizational managers in decision-making related to resource allocation and investment along digital supply chains.
[31]	“Lean Tools in the Context of Industry 4.0: Literature Review, Implementation and Trends”	Industrial Sector	explores the intersection of Industry 4.0 and Lean philosophy, addressing issues of inefficient digitalization within organisations.
[38]	“Which tools are needed to implement Lean Production in an Industry 4.0 environment? A literature review”	Business Sector	provide insights into how LP practices interact with Industry 4.0 technologies, shedding light on the tools currently employed by companies and the benefits they derive from their implementation.
[30]	“Exploring relationships between Lean 4.0 and the manufacturing industry”	Manufacturing	comprehensive understanding of Lean 4.0 and its implications for manufacturing industries. It aims to highlight the potential of Lean 4.0 technologies, such as the internet of things, artificial intelligence, three-dimensional printing, robotics, real-time data, cloud computing, predictive analytics, and augmented reality, in reducing waste and improving production processes.
[39]	“A Qualitative Analysis of Organisational Commitment in an Algerian Pharmaceutical Industry”	pharmaceutical industry	factors that hinder organizational commitment and understand their implications for organizational performance. The methodology involves conducting qualitative research, specifically a case study approach, to explore the complexities of organizational commitment within the Algerian pharmaceutical context
[40]	“Importance of Top Management Commitment to Organizational Citizenship Behaviour towards the Environment, Green Training and Environmental Performance in Pakistani Industries”	Manufacturing	understand how green training influences OCB-E and evaluate the impact of top management commitment on environmental performance and green training. The study hypothesizes that green training positively affects OCB-E and that top management commitment is associated with environmental performance.
[41]	“The Role of Frictions due to Top Management in Alliance Termination Decisions: Insights from Established Bio-Pharmaceutical Firms”	pharmaceutical industry	To provide insights into how decision-making processes within large firms influence the termination of research alliances and the role of top management teams in this process.

Although these notions are important in many sectors and places, the current research often neglects the viewpoints and experiences of companies in developing nations. The researcher therefore stands out for its niche focus on Lean 4.0 implementation in the pharmaceutical sector in Ghana, particularly emphasizing the role of top management commitment. This specificity distinguishes it from other articles Hence, future studies should aim to expand the area of investigation to include a wider array of businesses and geographic locations, including emerging economies such as Ghana.

### 2.4 The Lean 4.0 in pharmaceutical production

To increase operational efficiency in the manufacturing processes, the pharmaceutical sector primarily relies on the implementation of Lean 4.0 technology and lean concepts [42,43]. According to Ref. [44,45] the link between Industry 4.0 technology and Lean concepts is becoming more widely acknowledged in the pharmaceutical industry as a critical component for improving productivity, quality, and innovation. The subtle relationships between Industry 4.0 technologies—which are defined by automation, connectivity, and digitization—and Lean principles, which prioritize waste reduction and continual improvement [43].

Maximizing value while minimising waste is the notion at the heart of lean principles [46,47]. Lean approaches seek to maximise operational efficiency and increase product quality by creating a culture of continuous improvement, optimising processes, and getting rid of non-value-added tasks [48,49]. Lean concepts have been effectively used in the pharmaceutical industry to improve supply chain management, production procedures, and quality control, among other areas of manufacturing [44,50] The synergy between Lean principles and Industry 4.0 technologies lies in their shared goal of driving operational excellence and innovation. By integrating Lean methodologies with advanced digital technologies, pharmaceutical companies can unlock new opportunities for process optimisation, product customization, and supply chain visibility. For example, IoT-enabled sensors can monitor temperature and humidity levels in storage facilities [14,28,29].

#### 2.4.1 The interrelationships adopting lean 4.0 in pharmaceutical production

To increase operational efficiency in the manufacturing processes, the pharmaceutical sector primarily relies on the implementation of Lean 4.0 technology and Lean. Lean concepts and Industry 4.0 technology work together because they both want to promote innovation and operational excellence [[33], [51], [52], [53], [54], [55]]. Pharmaceutical firms can unleash new potential for supply chain visibility, product customization, and process optimisation by combining Lean approaches with cutting-edge digital technology [[56], [57], [58], [59]]. IoT-enabled sensors, for instance, may keep an eye on the humidity and temperature in storage facilities, guaranteeing the integrity of pharmaceutical items through the supply chain. In a similar vein, real-time production data analysis powered by AI algorithms may spot inefficiencies and recommend process upgrades that boost output while cutting waste [52,58,60,61]. The concept of mass customization in the pharmaceutical factory sets itself aside from Lean, whose main focus was the reduction of waste in mass production to improve lead time [33,62]. Another example of Industry 4.0 technology is the adaption of cyber-physical systems in the production and monitoring of drugs [60,61] This is a system which adapts the mixing of drugs based on real-time sensor monitoring to adjust the level of mixing to the properties of the current batch of raw materials [[63], [64], [65]], this contrasts with Lean, which has the aim of eliminating of human-based judgment in decision-making processes [59]. This is further contrasted by Lean's principle of mistake-proofing, which aims to prevent defects, often through simplicity and the use of low-technology inspection devices.

Moreover, by addressing some of the fundamental shortcomings of conventional Lean approaches, the implementation of Industry 4.0 technology enhances Lean concepts [[57], [58], [59]]. Industry 4.0 technologies provide automated data gathering, remote monitoring, and predictive analytics, which enhance the efficacy of Lean projects [58] According to Ref. [42,45,50] pharmaceutical firms may increase operational efficiency and agility by using digital technology to automate repetitive operations and support data-driven decision-making[48,66].

Notwithstanding the possible overlaps between Industry 4.0 technology and Lean concepts, there are still difficulties in integrating and putting them into practice [67]. One of the main obstacles is cultural resistance to change, which arises when Top management and staff members are reluctant to accept new working practices or technological advancements [68]. Furthermore, some pharmaceutical organisations find it difficult to undertake digital transformation efforts due to their complexity and the need for large expenditures in talent development and technological infrastructure[[69], [70], [71]].

There is a lot of promise for advancing innovation, productivity, and quality in the pharmaceutical industry due to the interaction between Lean concepts and Industry 4.0 technology[31,72]. Pharmaceutical companies can achieve new levels of competitiveness and sustainability in the changing healthcare landscape by utilizing Industry 4.0 technologies to digitise operations and automate workflows, and by leveraging Lean methodologies to streamline processes and foster a culture of continuous improvement. Nevertheless, achieving these advantages calls for organisational coherence, strategic leadership, and a dedication to lifelong learning and adaptability in the face of technological change.

#### 2.4.2 Divergences between lean principles and industry 4.0 technologies in pharmaceutical production

When examining the differences between Industry 4.0 technology and Lean principles [60,[72], [73], [74]], it's important to understand that although both seek to increase operational efficiency and spur innovation, they achieve these goals via different strategies and areas of concentrate First and foremost, Lean principles which have their roots in the Toyota Production System place a strong emphasis on waste reduction, ongoing development, and human respect [[75], [76], [77]]. Through the identification and elimination of non-value-added activities including overproduction, faults, and excess inventory, lean approaches prioritize process optimisation. Lean also encourages frontline staff to spot inefficiencies and participate in efforts to solve problems by fostering an environment of employee empowerment and engagement [53,77]. Conversely, Industry 4.0 technologies signify the amalgamation of digitization, automation, and data interchange, hence engendering "smart factories” and interlinked supply networks. Technologies like the Internet of Things (IoT), artificial intelligence (AI), big data analytics, and cyber-physical systems (CPS) are all included in Industry 4.0 [[76], [77], [78]]. Traditional industrial processes are being revolutionised by these technologies, which allow for real-time data collecting, predictive maintenance, and autonomous decision-making [79]. A significant distinction between Industry 4.0 technology and Lean principles is how each tackles process optimisation differently[80]. While Industry 4.0 technologies offer dramatic process transformation via digitalization and automation, Lean focuses on simplifying current processes and reducing waste through incremental improvements [79,81,82]. The emergence of Industry 4.0 technologies holds promise for upending conventional manufacturing paradigms via the facilitation of decentralised decision-making, self-governing production systems, and large-scale mass customization. Their differences also lie in how much emphasis they place on integrating technology and involving people [79].

Lean concepts place a high value on human-centric methods of problem-solving and decision-making, depending on the knowledge and ingenuity of frontline employees to spur ongoing development [82,83]. The integration of cutting-edge digital technology, such as robots and artificial intelligence (AI), to automate repetitive work and enhance human skills is the focus of Industry 4.0 technologies, in contrast [53,78]. While Industry 4.0 technologies raise worries about the possible replacement of human labour by automation and AI-driven systems, Lean encourages a culture of employee empowerment and involvement.

Furthermore, there are differences between Industry 4.0 technology and Lean principles in terms of organisational needs and implementation issues. A cultural transformation and an organisational commitment to continuous improvement are prerequisites for lean approaches, which often call for adjustments to performance management systems, communication protocols, and leadership styles [31,53]. The adoption of Industry 4.0 technologies, on the other hand, necessitates large expenditures in talent development, data governance, and technological infrastructure in addition to handling cybersecurity threats and privacy issues related to digital transformation projects. Moreover, the differences between Industry 4.0 technology and Lean principles emphasise the need for an integrated and comprehensive approach to manufacturing excellence [31,69,84].

### 2.5 Corporate performance

The effectiveness and efficiency of an organization in achieving its objectives manifest in the intricate concept of corporate performance. Success, a pivotal metric, is gauged through an array of financial and non-financial measures. As time has progressed, the understanding of corporate performance has evolved to encompass a broader spectrum of indicators, recognizing the multifaceted elements contributing to organizational success [85].

A noteworthy trend in evaluating company success is the growing acknowledgement of the importance of non-financial indicators. Traditionally, assessments of company success heavily relied on financial metrics such as profitability, return on investment, and market share. However, in recent years, there has been a rising awareness of the significance of non-monetary indicators, including customer satisfaction, innovation, social responsibility, and employee engagement [86]. This shift reflects a more holistic approach to gauging organizational performance that goes beyond traditional financial metrics.

### 2.6 The Upper Echelons Theory

Grounded in the psychological and socio-behavioural sciences, this theory posits that observable demographic characteristics of senior executives can serve as predictors of organizational outcomes. In essence, it serves as a bridge, connecting our understanding of managerial cognition at an individual level with its consequences at the organizational level [87]. The theory contends that the experiences, values, and personalities of senior executives profoundly shape their interpretation of the challenges they face. Consequently, these interpretations wield a significant influence over their decision-making processes and the strategic assessments they ultimately formulate. As a result, these decisions bear a substantial impact on the organization's trajectory and efficiency [87]. Therefore, this theory provides a profound understanding of how the dedication, traits, and strategic decisions of top management influence the adoption and effective implementation of Lean 4.0 in the pharmaceutical business of Ghana.

### 2.7 Resource-based view theory

A strategic management framework known as the Resource-Based View (RBV) hypothesis posits that companies can secure and sustain a competitive edge by distinctively leveraging their valuable resources. Introduced by Wernerfelt in 1984 and further refined by researchers like Barney in 1991, the Resource-Based View (RBV) underscores the role of organizational capabilities in establishing a sustained competitive advantage [88].

The core tenet of the Resource-Based View (RBV) asserts that a company's success is primarily determined by the resources it possesses and manages. These resources can be categorized as tangible, encompassing physical and financial assets, or intangible, including reputation, culture, knowledge, and skills. However, not all resources confer a lasting competitive advantage. Collins Christopher [88] proposed that for a resource to yield a sustained competitive advantage, it must exhibit four characteristics, referred to as the VRIN framework (Valuable, Rare, Inimitable, Non-substitutable).

Despite the wealth of literature on Lean 4.0 in various fields, there is a noticeable gap in understanding top management commitment to Lean 4.0 in pharmaceutical manufacturing and a lack of information regarding the correlation of Lean 4.0 with corporate performance. The need for strong commitment from top management in implementing Lean 4.0 in the pharmaceutical manufacturing industry is not well understood. Additionally, there is a clear dearth of knowledge addressing the intricate relationship between the adoption of Lean 4.0 and its impact on corporate performance. While numerous studies have explored Lean 4.0 and top management in different fields, there is a notable absence of research specific to the Ghanaian pharmaceutical manufacturing sector on the impact of top management commitment to Lean 4.0. To address these gaps, the following hypothesis was formulated.

### 2.8 Research gap analysis

In pursuit of understanding the dynamics of Lean 4.0 implementation and its impact on corporate performance, a comprehensive research gap analysis reveals critical areas where further investigation is warranted. This analysis aims to identify key knowledge gaps and inform future research directions to advance scholarly understanding and practical application in this domain.

Despite the acknowledged importance of top management commitment in driving organizational change and the successful implementation of Lean principles, there remains a notable gap in understanding the specific nature and extent of the relationship between top management commitment and Lean initiatives. Existing literature often emphasizes the general significance of leadership support but lacks detailed insights into how different dimensions of top management commitment (e.g., vision, resource allocation, communication) influence the adoption and effectiveness of Lean practices. Further research is needed to explore the mechanisms through which top management commitment translates into tangible outcomes in Lean implementation, such as improved process efficiency, employee engagement, and organizational culture.

Similarly, while the role of top management commitment in fostering technological innovation and digital transformation has been recognized, there is a gap in understanding its specific influence on the adoption and integration of Industry 4.0 technologies. Research in this area often focuses on the technical aspects of Industry 4.0 implementation, overlooking the critical role of leadership in driving organizational readiness, change management, and strategic alignment. Future studies should explore how different facets of top management commitment (e.g., strategic vision, investment decisions, cultural support) impact the adoption, utilization, and performance outcomes of Industry 4.0 technologies in various organizational contexts.

Despite growing interest in the synergies between Lean principles and Industry 4.0 technologies, there remains a gap in empirical research that systematically evaluates the combined impact of Lean and Industry 4.0 on organizational performance. While individual studies have highlighted the potential benefits of Lean or Industry 4.0 in isolation, few have comprehensively examined their complementary effects or trade-offs when implemented together. Future research should employ rigorous methodologies to assess the synergistic effects of Lean and Industry 4.0 initiatives on key performance indicators such as productivity, quality, flexibility, and innovation. Additionally, studies should consider contextual factors, organizational capabilities, and implementation strategies that may moderate or mediate the relationship between Lean, Industry 4.0, and performance outcomes.

Finally, there is a gap in understanding the specific pathways through which Lean 4.0 initiatives influence corporate performance outcomes. While theoretical frameworks and case studies suggest potential mechanisms linking Lean 4.0 practices to improved operational efficiency, quality, and competitiveness, empirical evidence is limited and fragmented. Future research should employ longitudinal designs and multilevel analyses to examine the causal relationships between Lean 4.0 adoption, intermediate process variables (e.g., supply chain integration, employee skills), and ultimate performance outcomes (e.g., financial performance, market share). Moreover, studies should explore the moderating effects of organizational context, industry characteristics, and external environmental factors on the relationship between Lean 4.0 and corporate performance.

Addressing these research gaps through rigorous empirical investigations is essential for advancing scholarly understanding and practical application of Lean 4.0 principles in organizational settings. By elucidating the complex relationships between top management commitment, Lean, Industry 4.0, and corporate performance, future research can provide valuable insights and actionable recommendations for organizational leaders, policymakers, and practitioners striving for excellence in operational management and strategic innovation.

### 2.9 Development of hypotheses

The hypotheses aim to elucidate the explicit connections among the commitment of senior executives, Lean methodologies, Industry 4.0 technology, Lean 4.0, and the overarching success of a company.

This concept emanates from the foundational principles of Lean management, emphasizing the reduction of inefficiencies, continual improvement, and the creation of value. These principles are pivotal drivers of corporate performance. Reinforcing this notion is the Resource-Based View (RBV) theory, asserting that a corporation can attain a competitive advantage by possessing internal resources that are valued, scarce, unique, and non-substitutable. When adeptly employed, Lean methodologies can function as critical assets, enhancing operational efficiency and ultimately elevating business performance. Contemporary literature substantiates this concept with substantial empirical support [[89], [90], [91]].

H1 Top management commitment has a positive effect on lean practices.

The Upper Echelons Theory posits that the strategic decisions and performance outcomes of an organization are directly influenced by the characteristics of its senior executives. Consequently, the commitment of senior executives emerges as a pivotal determinant in steering strategic initiatives, such as the adoption of Lean methodologies. Under the purview of this hypothesis, the organizational culture and strategic priorities may be shaped towards Lean principles, fostering waste reduction and process improvements, contingent upon the demonstration of commitment and active involvement by senior management [92,93].

H2 Top Management Commitment has a positive effect on Industry 4.0 technologies.

The hypothesis posits that the integration of Lean principles with Industry 4.0 technology, referred to as Lean 4.0, exerts a positive influence on company performance. This proposition aligns with the Resource-Based View (RBV) theory, asserting that organisations proficiently utilizing their internal resources can gain a competitive edge. Positioned as a valuable asset, Lean 4.0 amalgamates Lean techniques with Industry 4.0 technology and, when effectively implemented, holds the potential to enhance both operational and financial performance. Contemporary literature offers a nuanced discussion, presenting arguments both in favour and against this concept. Tortorella et al. [29] suggest that Lean 4.0 implementation might enhance operational effectiveness, thereby positively impacting business profitability. Additionally, research conducted by Rossini et al. [null]indicates that the adoption of Lean 4.0 methods resulted in improved operational effectiveness, customer satisfaction, and financial outcomes within Indian manufacturing companies. However, divergent perspectives exist. According to some researchers [29], while Lean 4.0 has the potential to enhance performance, its implementation may be intricate and disruptive, potentially leading to temporary decreases in performance.

H3a Lean practices have a positive role for lean 4.0

H3c Industry 4.0 has a positive role for Lean 4.0

H3b Lean 4.0 has a positive effect on corporate performance.

The proposed hypothesis posits that the implementation of Lean 4.0 methods yields a positive impact on business performance. Rooted in the core principles of Lean management, which prioritize the reduction of inefficiencies, continuous improvement, and the creation of value, this concept aligns with the Resource-Based View (RBV) hypothesis. According to RBV, a corporation can attain a competitive advantage by possessing internal resources that are valuable, rare, inimitable, and non-substitutable. Lean 4.0 approaches, when applied adeptly, emerge as crucial assets fostering operational efficiency and, consequently, elevating business performance. This hypothesis finds substantial empirical support in contemporary literature [14,28].

The integration of these theories facilitates Lean 4.0 adoption in the pharmaceutical industry within a developing country such as Ghana. Through the integration of various theoretical areas, scholars may clarify the intricate relationship that shapes the direction of Lean 4.0 projects, including strategic decision-making, organisational capacities, and individual-level elements. This multidisciplinary approach improves the theoretical knowledge of Lean 4.0 adoption and provides managers looking to promote innovation and continuous improvement in pharmaceutical manufacturing organisations with useful takeaways.

H4 Lean practices have a positive effect on corporate performance

H5 Industry 4.0 has a positive effect on corporate performance

**Fig. 1:**
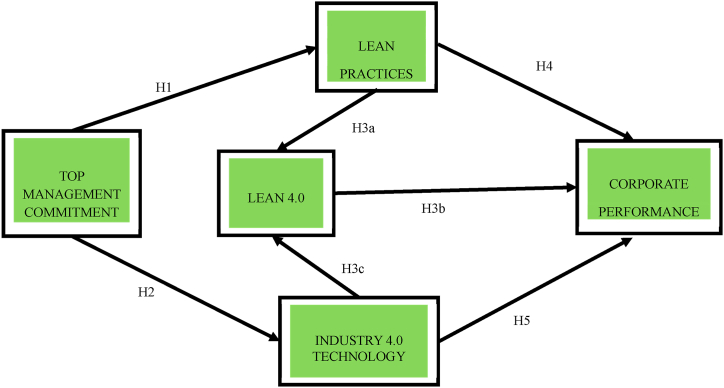
Theoretical model.

Fig. 1 illustrates the correlation between Hypothesis H1 and both Lean Practices (LP) and Top Management Commitment (TMC). Hypothesis H2 is interconnected with both TMC and Industry 4.0 Technology (I4.0). H3c is linked to both L4.0 and I4.0. H3b establishes connections with Lean 4.0 (L4.0) and CP. Hypothesis H4 is associated with both LP and CP, whereas Hypothesis H3a is linked with L4.0 and LP. Furthermore, Hypothesis H3b is correlated with L4.0 and CP. Consequently, the study has been structured to unfold in an ascending sequence.

## 3 Research methodology

This section provides a thorough examination of the research methodology employed in this study. The overarching objective is to fill the existing gap in research and, ultimately, to achieve a comprehensive understanding of the impact of top management commitment in implementing Lean 4.0 within the manufacturing sector of Ghana. To gather quantitative data and determine the appropriate industry for administering questionnaires, a well-designed questionnaire was employed. The subsequent discussion outlines the methods of data collection, offering transparency regarding the sources of data and the techniques employed for information gathering. This ensures clarity regarding the alignment of the data with the study's objectives and inquiries.

### 3.1 Research design

**Fig. 2:**
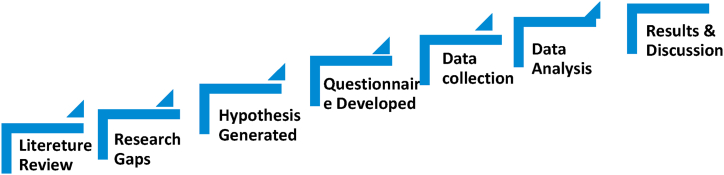
Outline of research process.

The research design employed for this study is explanatory, given the context. Explanatory research, also known as causal research, is utilized to discern and comprehend the cause-and-effect relationships between variables. This approach proves valuable when the study aims to elucidate the fundamental reasons or causes behind a phenomenon, as depicted in Fig. 2. It extends beyond mere description or exploration of connections, delving deeper into the determination of causation. This entails hypothesis testing, where the researcher proposes a cause-and-effect relationship and subsequently evaluates it using statistical methods.

### 3.2 Development of a survey questionnaire

A questionnaire, consisting of a predefined set of questions with specified answer choices, serves as a tool for collecting information. This survey was directed towards employees at various levels and across departments within the company. Employing a 7-point Likert Scale, respondents assessed practices on a scale ranging from 1 (Not at all) to 7 (Extremely). The detailed questionnaire, presented in the Appendix, comprised seven questions. To foster candid responses, personal inquiries such as name, age, gender, religion, ownership, and company name were intentionally excluded. Aligned with insights gleaned from the literature review, the questionnaire aimed to address highlighted issues and concerns, culminating in a summarized response.

### 3.3 Pilot study

In the pursuit of this research, a pilot study was undertaken. Initial inquiries were directed to academic experts, specifically excluding those in the Lean 4.0 field, to validate the questionnaire and gather sample responses. Following this, the questionnaire was circulated among a diverse group of industry experts for their scrutiny and recommendations regarding potential alterations. By incorporating the received suggestions and implementing necessary revisions, a comprehensive questionnaire was meticulously developed. This rigorous process ensured a thorough validation and refinement of the questionnaire, rendering it well-prepared for distribution.

### 3.4 Statistical tools

The initial step involved transferring data from Qualtrics to the Statistical Package for the Social Sciences (SPSS) software. This research utilizes two distinct software tools, namely SmartPLS (version 4) and IBM SPSS version 26, for comprehensive data analysis. SmartPLS is specifically designed for Partial Least Squares Structural Equation Modelling (PLS-SEM), a method employed to elucidate intricate multivariate interactions between observable and latent variables. In contrast, SPSS (Statistical Package for the Social Sciences) is a versatile program used for statistical analysis, offering a wide array of methods to examine data.

The data analysis in this research encompasses both descriptive and advanced statistical methodologies. To enhance the efficiency of the data analysis process, a unique code was assigned to each item in the dataset. This coding method ensures clarity and consistency throughout the analysis, facilitating easy referencing. Alongside coding, specifying the variable type was crucial for accurately describing the data. In this case, the variable type was designated as numeric, indicating that the variables held numerical data. The variables were assigned a width of 8, ensuring sufficient space for accommodating values, and the decimal precision was set to 0, indicating that the data did not require decimal values.

## 4 Data collection and analysis

### 4.1 Data collection

Pharmaceutical companies from various regions in Ghana were engaged in investigation, and data on the management of these industries were collected, etc. The questionnaire, designed in Qualtrics, aimed to enhance communication speed and user-friendliness for both filling out and collecting responses. Respondents were provided clear information about the purpose of data gathering and its intended use, with the option to decline or proceed with filling out the questionnaire. In total, two hundred online questionnaires were distributed to employees of pharmaceutical companies operating in Ghana. Out of the 200 distributed, 187 responses were received, resulting in an initial response rate of 93.5 %, reflecting the keen interest and willingness of the target population to participate in the study. After eliminating 6 responses, the useable response count stood at 181.

The subsequent crucial stage involved data entry and coding, emphasizing factors to ensure precise and effective data analysis. Initially, data from Qualtrics was imported into the Statistical Package for the Social Sciences (SPSS) program, facilitating analysis using various statistical methodologies. Each dataset was assigned a distinct code, providing a unique identity to each item. In addition to coding, specifying the variable type was essential for accurately representing the data. Here, the variable type was set as numeric, indicating that the variables held numerical data, with variable width adjusted to adequately accommodate values. The decimal precision was set to 0, signifying that the data did not require decimal values.

### 4.2 Preliminary data analysis

To enhance clarity and facilitate understanding, variables were allocated a defined range of values. In this case, a Likert scale ranging from 1 to 7 was employed, where a rating of 1 indicated "strongly disagree” and a rating of 7 indicated "strongly agree".

Table 2 illustrates job positions with a well-distributed representation, encompassing approximately 30 % in middle management, 27 % in senior staff, 21 % in first-level management, and 11 % in executive or entry-level positions. This diverse spread suggests the sample effectively captured views from various organizational levels, enhancing generalizability. Concerning areas of work, the largest share (31 %) was in production/manufacturing roles, followed by 20 % in purchasing/supply chain and 19 % in marketing/sales. The remaining portion was allocated to research and development, information technology, human resources, finance, and other departments. The diverse representation across organizational levels and functional areas enhances the reliability and applicability of the survey findings to the organization as a whole. Management can use these insights to gain a comprehensive understanding of employee perspectives and prioritize initiatives that address the needs and challenges across various job roles and departments. Detail survey data analysis has been provided in Appendix Section B and illustrated in Tables B1 to B5.

**Table 2:** Profile information.

Items		Frequency	Valid Percentage
Level of job position	Executive or senior management	19	10.5 %
	Middle management	54	29.8 %
	First-level management	38	21 %
	Intermediate or experienced (Senior staff)	49	27.1 %
	Entry level	21	11.6 %
Area of Work	R&D/Product Design & Development	14	8.4 %
	Purchasing/Supply Chain	36	19.9 %
	Production/Manufacturing	56	30.9 %
	Marketing & Sales	34	18.1 %
	Information Technology	9	5 %
	Human Resource Management	8	4.4 %
	Finance	19	10.5 %
	Other	5	2.8 %
Size of Organisation	Small (Less than 50 employees)	19	10.5 %
	Medium (Between 50 and 250 employees)	74	40.9 %
	Large (More than 250 employees)	88	48.6 %

### 4.3 Common method bias

The survey data may have common method bias since all of the dependent and independent variables are extracted from a single instrument (questionnaire) in a single phase. Experts and English language specialists worked together to design a well-crafted survey answer questionnaire that removes the CMB problem and ensures that no questions are unclear or double-barred. Since it is impossible to completely exclude CMB from survey replies, we statistically investigated and examined CMB-related problems. There is no CMB problem according to the results of the Hermans single factor test, which reveals that only 26 % of the variation is explained by the single component. Nevertheless, common latent factor (CLF) testing has been conducted since Hermans' single component test does not adequately guarantee the CMB removal. The results of the CLF analysis indicate that the CFA model with a common factor is not well-fitting, as shown by the unfit GFI 0.54, CFI 0.65, and RMSEA 0.12. This CLF analysis likewise showed that CMB had no bearing on the results of our investigation.

### 4.4 CFA

Table 3 presents the results of the Confirmatory Factor Analysis (CFA), examining the internal consistency, reliability, and convergent validity of each construct using statistical measures such as Cronbach's alpha (CA), Composite Reliability (CR), Average Variance Extracted (AVE), and individual item loadings. These results reinforce the systematic verification of the variables used in the research, thereby enhancing the credibility and reliability of the study.

**Table 3:** CFA results.

CFA					
Constructs		Factor Loadings	CA	CR	AVE
Lean 4.0	L4.01	0.86	0.87	0.91	0.62
	L4.02	0.81			
	L4.03	0.56			
	L4.04	0.79			
	L4.05	0.83			
	L4.06	0.84			
Top Management Commitment	TMC1	0.83	0.87	0.90	0.57
	TMC2	0.78			
	TMC3	0.79			
	TMC4	0.79			
	TMC5	0.84			
Lean Practices	LP1	0.83	0.88	0.91	0.65
	LP2	0.75			
	LP3	0.73			
	LP4	0.85			
	LP5	0.76			
	LP6	0.90			
Industry 4.0	I4.01	0.80	0.89	0.92	0.57
	I4.02	0.59			
	I4.03	0.65			
	I4.04	0.81			
	I4.05	0.88			
Corporate Performance	CP1	0.80	0.87	0.90	0.52
	CP2	0.80			
	CP3	0.52			
	CP4	0.74			
	CP5	0.66			
	CP6	0.65			
	CP7	0.72			
	CP8	0.84			

For the "Lean 4.0″ construct, factor loadings range from 0.56 to 0.86. Notably, the Composite Reliability (CR) for this construct is recorded at 0.91, surpassing the conventional threshold of 0.7, indicating robust internal consistency. The Cronbach's Alpha (CA) for this construct is 0.87, signifying a good level of internal consistency. Additionally, the Average Variance Extracted (AVE) score of 0.62 exceeds the suggested threshold of 0.5, demonstrating excellent convergent validity ∼ B′08. The dependability and validity of the Lean 4.0 architecture provide management with significant insights into the efficacy and coherence of Lean 4.0 projects in pharmaceutical production. By acknowledging the strong internal consistency, a satisfactory degree of internal consistency, and outstanding convergent validity of Lean 4.0 methods, management can make well-informed choices and investments to promote ongoing progress and innovation inside the organisation.

The "Top Management Commitment” construct exhibits factor loadings ranging from 0.78 to 0.84, showcasing high dependability with a CR of 0.90. The "Lean Practices” construct has factor loadings ranging from 0.73 to 0.90, with a coefficient of restitution (CR) of 0.91, emphasizing its high reliability. For the "Industry 4.0″ construct, factor loadings range from 0.59 to 0.88, with a CR of 0.92, highlighting exceptional dependability. The coefficient alpha (CA) with a value of 0.89 further underscores the impressive level of internal consistency. The "Corporate Performance” construct displays factor loadings ranging from 0.52 to 0.84, with a coefficient of reliability of 0.90, indicating a high level of dependability. Additionally, it has a coefficient alpha (CA) of 0.87, further reinforcing its reliability.

The dependability and accuracy of these concepts provide senior executives with significant standards for evaluating the efficiency of Lean 4.0 implementation and its influence on company performance. To successfully lead organisational change initiatives and generate sustainable development in the pharmaceutical business, senior executives must acknowledge the importance of top management commitment, Lean methods, Industry 4.0 technology, and corporate performance indicators.

Table 4 presents a comprehensive analysis of the Heterotrait-Monotrait (HTMT) values, with each combination of components showing ratios consistently below the 0.90 threshold. HTMT analysis ensures there is no discriminant validity concern in the study. Specifically, the HTMT values for the various relationships are provided in Table 4. Notably, the highest HTMT ratio is 0.77, observed between Lean 4.0 Practices and Top Management Commitment. The HTMT analysis provides robust statistical support, affirming that each construct in this study possesses distinct characteristics. None of the constructs exhibit redundancy or are indistinguishable from each other, reinforcing the notion that top management commitment, lean practices, lean 4.0 practices, industry 4.0 technology adoption, and corporate performance each contribute unique variances to the study.

**Table 4:** Result of Heterotrait-Monotrait ratio (HTMT) Test.

	CP	I4.0	L4.0	LP	TMC
**CP**	0.72				
**I4.0**	0.74	0.75			
**L4.0**	0.77	0.67	0.79		
**LP**	0.72	0.53	0.80	0.61	
**TMC**	0.73	0.78	0.77	0.72	0.71

Table 3 displays the results of the Confirmatory Factor Analysis (CFA), providing a detailed overview of lower-order constructs. Key parameters such as factor loadings, Cronbach's Alpha (CA), Composite Reliability (CR), and Average Variance Extracted (AVE) are systematically presented for clarity.

Within the "Lean 4.0″ construct, factor loadings range from 0.56 to 0.86. Significantly, the Composite Reliability (CR) for this construct is recorded at an impressive 0.91, surpassing the conventional threshold of 0.7 and underscoring robust internal consistency. The Cronbach's Alpha (CA) for "Lean 4.0″ is 0.87, further affirming the internal reliability of the items. These results collectively support the validity and reliability of the "Lean 4.0″ construct, reinforcing its robustness in capturing the intended dimensions. The strong internal consistency, internal reliability affirmation, and support for validity and reliability highlight the success and coherence of Lean 4.0 activities in pharmaceutical production. By acknowledging these significant discoveries, management can make well-informed choices and strategic investments to maximise the benefits of Lean 4.0 principles for ongoing development and long-term growth inside the organisation.

### 4.5 SEM

**Fig. 3:**
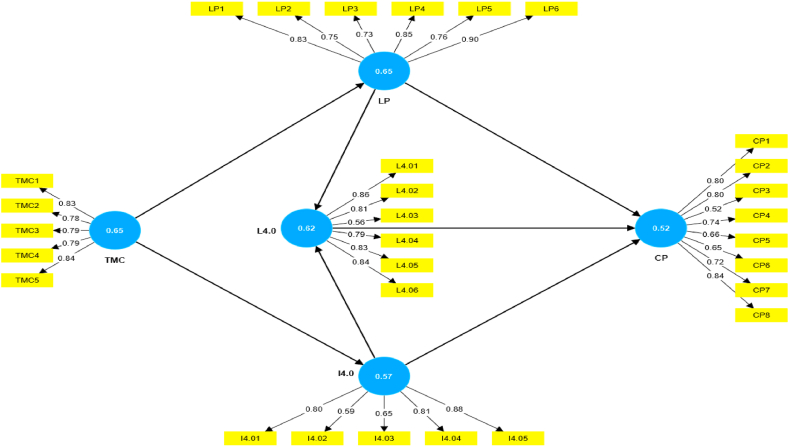
Sem diagram.

Fig. 3 shows SEM diagram, while Table 5 shows the interconnections within the structural framework. A positive coefficient in the TMC and LP relationship signifies a direct correlation—higher TMC corresponds to increased LP. The substantial T-value of 17.47 underscores the statistical significance, standing several standard deviations away from zero. This significance is reinforced by the P-value of 0.00, confirming its statistical robustness. The linkage between TMC and Industry 4.0 (I4.0) boasts a robust positive correlation, with a coefficient of 0.78 suggesting the united change in TMC has produced a 0.78 change in Industry 4.0. This suggests that an expansion in TMC yields a similarly positive impact on I4.0. Conversely, a negative coefficient of −0.09 characterizes the link between Lean Practice (LP) and Lean 4.0 (L4.0). The influence of Lean 4.0 (L4.0) on Corporate Performance (CP) reveals a positive albeit moderately low coefficient of 0.17 and significant at 0.09 level of significance. The coefficient 0.17 suggests that the unite change in L4.0 has produced a 0.17 change in Industry CP. This indicates a correlation between CP increase and L4.0 rise, though not as pronounced as in other demonstrated connections. An assessment of the strength and significance of these relationships validates the proposed hypotheses against the collected data. The NFI value of 0.82 suggests a moderate fit of the model, exceeding the common threshold and implying a good alignment of the model with the data. The square multiple correlation R2 values of every factor are healthy suggesting a good number of explanations of variance by the preceding factors. These results emphasise the significance of strong leadership from top management in enhancing organisational performance, promoting innovation, and effectively adapting to the changing environment of lean methods and Industry 4.0 technology. Through comprehending the interconnectedness and disparities among these elements, management may formulate strategic choices to maximise efficiency, bolster competitiveness, and attain long-lasting expansion.

**Table 5:** Structural Framework Interconnections

Path	Path Coefficients	T-value	P-value
Direct Effects			
TMC→ LP	0.82	17.47	0.00
TMC → I4.0	0.78	21.78	0.00
LP → L4.0	−0.09	2.11	0.03
L4.0 → CP	0.17	1.70	0.09
I4.0 → L4.0	0.52	4.04	0.00
LP → CP	0.27	3.51	0.00
I4.0 → CP	0.20	2.15	0,04

Coefficient of Determination				
	CP	I4.0	L4.0	LP
R^2^	0.80	0.61	0.77	0.68
Δ R^2^	0.80	0.61	0.77	0.68
F^2^ Effect				
	CP	I4.0	L4.0	LP
I4.0	0.04		0.60	
L4.0	0.26			
LP	0.11		0.09	
TMC		1.60		2.12
Model Fit Indicators				
SUMMER	0.07			
d_ULS	2.26			
d_G	1.34			
Chi-square	1213.30			
NFI	0.82			

According to Table B6 of appendix B, the examination of statistical significance and direction of relationships reveals that the hypothesis positing a positive impact of Lean 4.0 (L4.0) on Corporate Performance (CP) was not supported (H3b, L4.0 → CP, 1.70, 0.17; P > 0.05). This suggests that the transition to Lean 4.0 may not immediately translate into enhanced corporate performance. The absence of quick evidence supporting the theory that connects Lean 4.0 to corporate performance emphasizes the need for organisations to embrace a methodical and strategic approach while implementing Lean 4.0. To optimise the long-term advantages of Lean 4.0 initiatives and promote sustainable organisational performance, management should focus on managing expectations, recognizing delayed effects, implementing comprehensive performance evaluation methods, embracing ongoing improvement, and ensuring strategic alignment and integration.

## 5 Discussion

The implementation of Lean Practices (LP) demonstrates a significant influence from Top Management Commitment (TMC), as indicated by the data (H1, TMC → LP, 17.47, 0.82; p < 0.01). This correlation is consistent with the theoretical framework of the Upper Echelons Theory, suggesting that the strategic direction of an organization is shaped by the values and commitments of its top management [93,94]. The connection is further supported by the Resource-Based View (RBV) [95]. Empirical evidence underscores the significance of TMC (Top Management Commitment) in the successful adoption of lean practices [96,97]. Furthermore, effective execution requires translating TMC into actionable plans, supported by resources and employee involvement [98,99].

The evidence strongly supports the notion that Top Management Commitment (TMC) positively influences the acceptance and effective application of Industry 4.0 technologies (I4.0) (H2, TMC → I4.0, 21.78, 0.78; p < 0.01). The Upper Echelons Theory posits that an organization's strategic choices, particularly concerning technology utilization, mirror the attributes and commitment of its senior leaders [100]. This theory acknowledges the profound impact of these technologies and the significant transformations they introduce to an organization's operations and culture [101].

The commitment to the Resource-Based View (RBV) plays a crucial role in shaping the overall strategy, facilitating resource allocation, and nurturing a culture that embraces technological innovation [92,93,94]. However, it is important to note that a proficient labour force, a supportive culture, and an environment conducive to innovation are equally essential components [92]. Lean Practices (LP) exert a positive impact on Lean 4.0 (L4.0), although the magnitude of this effect is relatively moderate (H3a, LP → L4.0, 2.11, −0.09; p < 0.05). The perpetual refinement of lean processes over time culminates in the creation of highly valuable assets that adeptly facilitate the integration of Industry 4.0 technologies, thus facilitating the evolution toward Lean 4.0 [null].

Existing research underscores that organisations proficient in lean methodologies are more likely to effectively assimilate Industry 4.0 technology, marking significant progress towards Lean 4.0 [102,103]. However, it is crucial to acknowledge the inherent challenges in aligning certain lean principles with the intricacies of Industry 4.0 technologies [104]. This transformative journey extends beyond mere technological adoption; it necessitates the adaptation and evolution of lean principles to synergize with the capabilities and potentials inherent in Industry 4.0.

Industry 4.0 technologies significantly contribute to the positive development of Lean 4.0 (H3c, I4.0 → L4.0, 4.04, 0.52; p < 0.01). This symbiotic relationship is best comprehended through the lens of the Resource-Based View (RBV), which posits unique and strategic resources as pivotal to achieving a competitive advantage. In this context, Industry 4.0 technologies are regarded as strategic resources that augment a firm's capacity to successfully implement Lean 4.0 [105]. Consequently, while Industry 4.0 technologies play an instrumental role in the evolutionary trajectory toward Lean 4.0, their effective integration mandates strategic planning, alignment with lean principles, and adept management of potential complexities. Only through such comprehensive approaches can organisations fully realize the multifaceted benefits stemming from the convergence of Industry 4.0 and Lean 4.0.

The hypothesis proposing a positive impact of Lean 4.0 (L4.0) on Corporate Performance (CP) was not substantiated (H3b, L4.0 → CP, 1.70, 0.17; P > 0.05), suggesting that the transition to Lean 4.0 may not yield an immediate enhancement in corporate performance. This finding highlights the intricate nature of implementing Lean 4.0, a fusion of lean practices with Industry 4.0 technologies. The absence of an immediate positive effect on CP indicates that while Lean 4.0 holds the promise of operational efficiencies and waste reduction, these advantages may necessitate time to materialize into quantifiable improvements in corporate performance. This process could be intricate and resource-intensive [29], emphasizing the importance of recognizing the enduring benefits of Lean 4.0 in augmenting corporate performance over the long term.

The positive impact of Lean Practices (LP) on Corporate Performance (CP) is distinctly observed (H4, LP → CP, 3.51, 0.27; p < 0.01). This result resonates with the foundational tenets of lean management, emphasizing waste reduction, continuous improvement, and value creation—fundamental elements for augmenting corporate performance. However, the efficacious implementation of lean practices transcends the mere adoption of tools or methodologies; it necessitates a cultural transformation within the organization. This cultural shift propels the establishment of a continuous improvement mindset, engaging all employees in the identification and elimination of waste within their respective areas of work [106].

The research substantiates the hypothesis that Industry 4.0 (I4.0) technologies exert a positive influence on Corporate Performance (CP) (H5, I4.0 → CP, 2.15, 0.20; p < 0.05). These technologies facilitate heightened operational efficiency, data-driven decision-making, and improved customization—core elements contributing to enhanced corporate performance. The integration of big data analytics furnishes valuable insights into customer behaviour and market trends, empowering pharmaceutical manufacturing to make well-informed strategic decisions. Nevertheless, companies must ensure the establishment of suitable infrastructure and provide adequate training for their workforce to effectively harness the potential of these new technologies.

### 5.1 Success Factors of Top management commitment and implementation

#### 5.1.1 Success Factors

This research is crucial and timely as Lean 4.0 is still in its infancy in Ghana and there is a dearth of information on it [107,108] Identifying the right implementation strategies and the factors that will lead to successful implementation is pivotal at this stage as it will shape the future of Lean 4.0 in Ghana [82,83,109] Lean 4.0 implementation requires many technical changes and advancements in the present working system [82,83] When Top management gets dedicated to anything, it reflects on its success and positive outcome.

Implementing Lean 4.0 in the pharmaceutical industry, with the supervision of top management, may result in heightened efficiency, enhanced quality control, reduced costs, greater regulatory compliance, higher innovation, superior supply chain management, and heightened satisfaction among consumers. These results eventually support the industry's objective of delivering secure, efficient, and cost-effective drugs to patients worldwide [[110], [111], [112], [113]]. According to Ref. [114,115] top management embracing Lean 4.0, pharmaceutical companies can unlock a multitude of benefits across various facets of their operations, ultimately leading to increased efficiency, improved quality control, cost reduction, enhanced regulatory compliance, increased innovation, better supply chain management, and enhanced customer satisfaction. An important benefit of Lean 4.0 is its capacity to optimise production procedures in pharmaceutical facilities. By integrating advanced Industry 4.0 technologies like automation, robots, and data analytics with Lean approaches, top-level executives may streamline production processes, reduce downtime, and improve resource efficiency. Consequently, the production of medications may be expedited and made more accurate, guaranteeing prompt distribution of therapies to individuals requiring them [115].

Furthermore, Lean 4.0 prioritises the utilization of data and analytics to oversee and regulate production processes, hence improving quality control protocols[116]. Using up-to-the-minute observations on production activities, senior executives may promptly identify and resolve any deviations or irregularities in pharmaceutical items, guaranteeing that only secure and efficacious pharmaceuticals are delivered to customers. Ensuring patient safety not only protects but also improves the reputation and credibility of pharmaceutical companies in the market [56,117]. Lean 4.0 not only enhances efficiency and quality but also empowers top management to achieve substantial cost savings in the pharmaceutical production process [82]. Pharmaceutical firms may efficiently decrease manufacturing costs by detecting and removing waste across the value chain, optimising resource utilization, and minimising needless spending. Consequently, this leads to a reduction in the cost and availability of pharmaceuticals for patients, thereby facilitating fair and equal distribution of healthcare[115].

Moreover, Lean 4.0 is crucial in guaranteeing adherence to regulations in the heavily regulated pharmaceutical sector [118]. To comply with regulatory standards, top management must implement rigorous quality control systems and standardised procedures to ensure product safety and quality[82,118,119] Pharmaceutical firms may enhance their compliance efforts, ensure precise record-keeping, and reduce the risks associated with non-compliance by adopting Lean 4.0. This will ultimately protect the health and well-being of patients. Lean 4.0 promotes a culture of continuous improvement and innovation in pharmaceutical organisations, going beyond only operational efficiency and regulatory compliance [119]. Through the delegation of authority to workers at all hierarchical levels, top management may effectively stimulate innovation in the areas of medication discovery, formulation, and delivery. This facilitates the development of novel and enhanced pharmaceuticals that target unfulfilled medical requirements and enhance patient results. In addition, through the optimisation of inventory management, reduction of lead times, and enhancement of logistics procedures, top management can guarantee a dependable supply of medications to patients around the globe [42]. This improves the robustness and flexibility of pharmaceutical supply chains, allowing for prompt adjustments to evolving market demands and healthcare requirements. The primary objective of Lean 4.0 in the pharmaceutical industry is to ensure the timely provision of superior-quality pharmaceuticals to patients[82]. Through the implementation of strategies that improve efficiency, quality, and innovation in all areas of operations, senior management may increase customer satisfaction and instil confidence in their goods. Consequently, this results in improved health outcomes for patients and strengthens the pharmaceutical industry's dedication to enhancing the delivery of healthcare worldwide [82,118,120].

#### 5.1.2 Implementation of lean 4.0

**Fig. 4:**
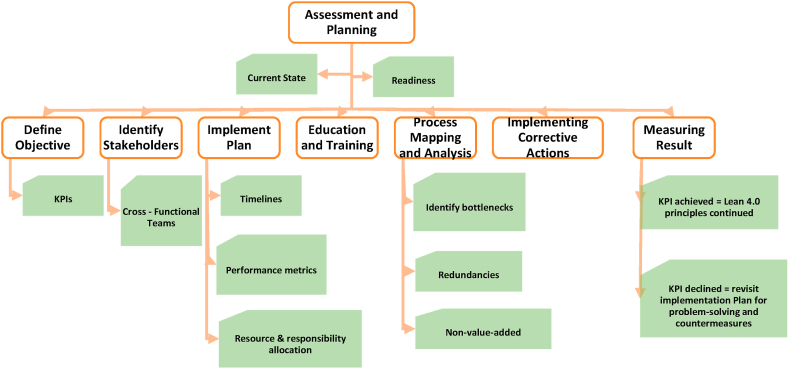
Implementation plan of lean 4.0.

The application of Lean 4.0 in the pharma sector is a completely novel concept [30]. This Industry 4.0-supported lean approach is expected to enhance the smooth functioning of the pharma business, obtaining better productivity, quality, and reducing operational costs. Large-scale capital investment, fragmented supply chain network, strict regulatory compliances, and extensive customer base make the pharma industry suitable for implementing this advanced concept of lean. Presently, pharmaceutical industries are facing tremendous pressure for high-quality and compliant products at a lower price. In such a scenario, Lean 4.0 can offer them a better solution [42,118]. According to Ref. [121] complex and bulky raw data and extensive research, the traditional lean approach lags in handling these data and offering a pragmatic solution. Therefore, the pharma sector being analytical has high potential for industry 4.0 because it can make use of the large data generated by different processes, convert it into information, and support the decision-making process more efficiently [122,123]. The system of Track and Trace supported by a secure system of product identification and authentication will identify every product input at various stages, ensuring the safety and quality of the product [123,124], as outlined in Fig. 4, are the stages of implementing Le.

System implementation is a large project, and it requires clear objectives to ensure the project's success[109] The initial stage is Assessment and Planning, this goal is to evaluate the readiness for Lean 4.0, and a clear understanding of the current state of operations within the organization must be established. This can be done through value stream mapping. Value stream mapping is a simple-structured, visual means to analyze the flow of information and materials in an action (or 'value stream') with the idea of exposing waste and throughput time[125,126], with a comprehensive assessment of current processes, workflows, and challenges within the pharmaceutical manufacturing environment. Extremely important as the processes can be classified into one of three categories value-added, non-value-added but necessary, and pure waste[127]. This is to assess whether the process carries out its intended purpose to add value. With this data, it is possible to identify why some areas of the company may need to be improved, changed or in some cases removed[123,125,128].

Define clear objectives and goals is the next stage, to identify potential areas for improvement such as improving efficiency, reducing waste, enhancing quality, and increasing flexibility [125,126,129,130]. Identify key stakeholders and establish a cross-functional team this is the next stage to lead the implementation process. This team bring on board internal experiences in each department and it aids in the implementation process. According to Ejsmont et al. [42] & Ilangakoon et al. [118], the planning team needs to have a good understanding of what Lean 4.0 is and what it can deliver. The next stage is the development detailed implementation plan, including timelines, resource allocation, and performance metrics to track progress. One of the most important aspects of planning a project is to develop a realistic timeline to track progress against expected outcomes. Changes do not happen overnight, and a good plan will establish the path from the current state to the desired future state, with clear expectations of the time required at each stage. The timeline will assist in calculating the benefits of the project, by knowing when the changes are expected to occur [131,132] this can be 12–18 months on the journey' everything leading up to that future state is divided into a sequence of 3–6 month. It also provides a benchmark for measuring the rate of change and helps keep the project on course and avoid drifting back into old practices[131], KPIs (Key Performance Indicators) are then set to ensure that these goals are met, the use of SMART (Specific, Measurable, Agreed upon, Realistic, Time-based) goal setting is encouraged here. This means defining the goals to be as specific as possible with a way to measure their attainment and the time frame in which they should be achieved [133,134]. The implementation plan must also cover Allocating Responsibilities and Roles to avoid the occurrence of ambiguities and potential confusion that jeopardise the clear understanding of team members [135].

Education and Training is the next stage, providing education and training on Lean 4.0 principles and methodologies to all employees involved in the implementation process. The most vital condition for the achievement of Lean 4.0 in the medical services area is to have all staff comprehend the essentials of Lean 4.0 and the significance of innovation [30,136]. The initial step in addressing this is to foster awareness of Lean 4.0 and how it differs from previous generations of Lean. Offer specialized training workshops and seminars tailored to different roles and responsibilities within the organization, such as production staff, quality control personnel, and management teams with a focus on the practical skills and mindset changes required for Lean 4.0 implementation. This training must foster a culture of continuous learning and improvement by encouraging employee participation.

Process Mapping and Analysis is the next stage, it is important that performance is monitored, in terms of efficiency and quality and compared with the original objectives and KPI developed, when performed regularly, KPI performance reviews will highlight areas of strength and weakness in comparison to the previous state and objectives. If a KPI is showing improvement, the Lean 4.0 principle states to continue the action that is causing that improvement. KPIs showing decline or no change in comparison to the original state need specific problem-solving and countermeasures to achieve improvement towards the objective [46,137,138]. An effective practice for managing KPIs is to plan the expected improvement targets into a "KPI storyboard” which will give a visual representation of the objectives and expected performance in all areas of the business. Identify bottlenecks, redundancies, and non-value-added activities that can be targeted for improvement. Analyze data and metrics to understand performance trends, identify root causes of problems, and prioritize improvement opportunities. It may be necessary to look at both the intangible and tangible results of a project to find a compromise that serves the future value stream [139].

The next stage is Implementing Corrective Actions, an essential part of Lean 4.0 philosophy is that change is an ongoing process. After monitoring and identifying that a process is deviating from the ideal state, corrective action must be taken. The aim is to 1) understand what went wrong, 2) eliminate the root cause, and finally 3) standardize methods of preventing re-occurrence [46,137,138]. During this process, cross-functional teams will be utilized to utilize a broad base of expertise to ensure effective problem resolution. In the short term, simple countermeasures may be put in place to contain the problem while a longer-term solution is identified and implemented.

Measuring the Result is the next step to consider, measuring the success of any Lean 4.0 implementation is difficult, but it is crucial. Improvement strategies of any sort are usually implemented to make positive changes, and it is necessary to determine whether or not these changes are happening [109,125,126]. In the case of Lean 4.0 in the pharmaceutical sector in Ghana, it is assumed that the overall goal is to make improvements to the existing lean implementation to solve the current problems being faced. This allows for a comparison to be made on the current situation, using evidence-based practice to see whether or not the implementation has been a success. This comparison is an important determination of success, as it allows changes to be shown tangibly. Without a clear comparison, it is difficult to say whether changes were successful or whether they made things worse. Stepwise improvement also allows for adjustments to be made at each stage, helping to minimize the risk of things going wrong.

The final stage in the Lean 4.0 process is to make adjustments and improvements to their current state based on the evaluation results [140,141]. This is a continuous loop which is designed to aid companies in making data-driven decisions which lead to step-change improvements to operations and help sustain the change process [46,133,137,138]. It is critical companies do not bypass this stage and move directly to the first step in implementation. This will result in no real improvements being made as there will be no evidence that the changes have a positive or negative effect on the process. When this happens, top management needs to review the objectives to make adjustments and improvements based on evaluation results.

## 6 Research implications

### 6.1 Managerial implications

The study delineates a comprehensive strategy for the successful implementation of Lean 4.0 transformation in pharmaceutical management practices. This strategic plan encompasses nuanced guidance on key elements such as strategic commitment, resource allocation, technology investments, and change management. The explicit focus on top management commitment and its interplay with lean principles and Industry 4.0 provides actionable insights for CEOs and leadership teams to adeptly champion this transformative agenda throughout their organisations. In practical terms, supply chain managers can leverage the research findings to assess process compatibility, strategically integrate technology at various stages, and strike a balance between individual advancements and the development of digitally interconnected systems. Overall, this research furnishes valuable guidance for navigating the intricate yet rewarding path of Lean 4.0 implementation.

### 6.2 Theoretical implications

According to UET, the adoption of Lean 4.0 can be seen as a reflection of the top executives' characteristics, such as their experiences, values, personalities, and cognitive styles. These characteristics influence how the top executives perceive, interpret, and respond to the strategic situations and opportunities that arise from the digital transformation of the manufacturing industry. Therefore, the role of top management commitment is to align the top executives' individual preferences, beliefs, and attitudes with the organizational goals, culture, and processes that can support the adoption and implementation of Lean 4.0. This study can help highlight the role of top management commitment in facilitating and supporting the adoption of Lean 4.0 in the pharmaceutical industry, such as the provision of strategic vision, leadership, resources, training, and incentives for the employees and partners. According to RBT, the adoption of Lean 4.0 can be seen as a strategic decision to acquire and leverage the digital resources and capabilities that can enhance the firm's operational performance and sustainability. However, the availability and affordability of these resources and capabilities may vary across firms and industries, depending on the level of competition, regulation, and innovation. Therefore, the role of top management commitment is to provide the strategic vision, leadership, resources, and incentives for the firm to invest in and utilize the digital technologies and practices that can create value for the firm and its stakeholders.

This study contributes significant theoretical insights, enriching our understanding of Lean 4.0 implementation and the pivotal determinants influencing its acceptance. The empirical data, derived from a relatively underexplored context, fills critical gaps in knowledge, making a substantial contribution to academic literature. The findings robustly support established hypotheses regarding the impact of strategic resources and top management commitment on driving organizational change. However, beyond unravelling intricacies in Lean 4.0 adoption, this research extends theoretical perspectives to encompass nuanced aspects of change management, cultural influences, and performance evaluation that are still evolving. In essence, it not only provides a robust foundation for generating fresh theoretical ideas but also lays the groundwork for new frameworks and research trajectories focused on Lean 4.0 implementation across diverse industrial and geographical landscapes.

### 6.3 Research contribution

#### 6.3.1 Contribution to the current industrial pharmaceutical scenario

This study significantly advances the current state of the industrial pharmaceutical industry by offering valuable insights into the implementation of Lean 4.0 in the pharmaceutical manufacturing sector of Ghana. The research evaluates the significance of top management commitment in the context of adopting Lean 4.0. This is of utmost importance as senior management acts as the catalyst for organisational change and ensures the effective execution of innovative methodologies such as Lean 4.0. Providing valuable insights into the impact of Lean 4.0 implementation on pharmaceutical production in Ghana through an assessment of its overall effectiveness and achievements. Companies desiring to implement or improve Lean 4.0 methodologies must possess this understanding. Hence ensures a rigorous approach to data collection and analysis through the implementation of a survey-based explanatory quantitative research design, guided by a positivist paradigm. This method improves the validity and dependability of the conclusions, as well as their applicability to academics and business professionals. A comprehensive analysis of the collected data is feasible through the implementation of sophisticated data analysis tools, including SmartPLS and IBM SPSS version 26. This enables a comprehensive examination of the correlations between various Lean 4.0 implementation-related factors and the results it has on the performance of an organisation. Comprehending Lean 4.0 integration underscores the importance of meticulous administration in the convergence of lean methodologies and Industry 4.0 technology. Pharmaceutical companies seeking to incorporate lean principles and state-of-the-art technologies into their processes will discover this information to be advantageous.

The study underscores the benefits that lean concepts and Industry 4.0 technology offer on the performance of organisations, notwithstanding the lack of immediate increases in profitability or productivity. This underscores the enduring benefits of adopting Lean 4.0 and inspires organisations to persevere in the face of initial challenges. For that reason, significantly advances the field of pharmaceutical production by providing evidence-based insights into the implementation and consequences of Lean 4.0. It catalyzes additional research in this domain and provides insight to policymakers, industry professionals, and scholars regarding the critical factors that influence the effectiveness of Lean 4.0 initiatives.

#### 6.3.2 Contribution towards technological transformation

Several strategic stages are required to transition from the current transformative potential to the seamless incorporation of technologies:

Determine Critical Technologies: Understand the technologies that possess the capacity to revolutionise your industry. This may involve blockchain, artificial intelligence, machine learning, robotics, or the Internet of Things (IoT). Evaluate the potential of these technologies to optimise organisational processes, boost productivity, and enhance decision-making.

Develop a Clear Roadmap: Formulate an unambiguous strategic plan delineating the manner and timing of the integration of said technologies into the workflow. Establish a hierarchy of technologies according to their potential impact and implementation feasibility. Establish attainable benchmarks and schedules to monitor advancement.

Invest in Training and Infrastructure: Guarantee that your organisation possesses the infrastructure required to facilitate the integration of these technologies. This may entail implementing software solutions, upgrading hardware, and establishing secure communication and data storage systems. Furthermore, Top management must ensure that employees have access to training and upskilling opportunities to utilize these technologies effectively.

Encourage a Culture of Innovation: Cultivate an environment that promotes and supports experimentation and innovation. Form cross-functional teams with the specific objective of investigating novel technologies and pinpointing potential areas for enhancement. Promote interdepartmental cooperation and engage external collaborators in collaborative efforts to capitalise on a wide range of expertise and perspectives.

Considerations of a regulatory and ethical nature: Comprehend the ethical and regulatory ramifications that accompany the implementation of novel technologies. Keep abreast of pertinent legislation and regulations that pertain to the protection of intellectual property rights, data privacy, and security. Establish protocols and policies to guarantee ethical and compliant technology usage.

Adapt and Monitor: Evaluate the impact of newly implemented technologies on your organisation on an ongoing basis. Seek input from stakeholders, customers, and employees to identify potential areas for adaptation and enhancement. Anticipate and adapt your technological strategy to evolving market dynamics and emergent opportunities flexibly. It is imperative to embrace agility and flexibility to effectively respond to market shifts and technological advancements. Develop a mindset that prioritises ongoing improvement and demonstrates a readiness to adjust or pivot as necessary. Maintain awareness of developing trends and proactively investigate novel prospects for expansion and innovation. By adhering to the prescribed procedures, organisations can proficiently leverage the revolutionary capabilities of technologies and effortlessly integrate them into their functioning, thereby fostering enduring expansion and a competitive edge in the era of digitalization.

### 6.4 Unique features of the study

Through its specific emphasis on the pharmaceutical sector in Ghana, this study offers valuable insights into a context that may have eluded attention in prior scholarly investigations. The recognition of this contextual significance is vital to comprehending the distinct obstacles and prospects encountered by pharmaceutical enterprises in Ghana, specifically about the implementation of Lean 4.0 methodologies. The research investigates the incorporation of Industry 4.0 technologies into Lean principles, an innovative concept within the pharmaceutical industry. Through an analysis of the potential synergy between these two frameworks, this study provides practical implications that can be leveraged to improve pharmaceutical manufacturing efficiency and quality. The study highlights the critical importance of leadership dedication in the successful integration of Lean 4.0 principles within the pharmaceutical manufacturing sector of Ghana. This underscores the significance of support and buy-in from leadership to propel innovation and organisational change. The research study utilizes an all-encompassing methodology, incorporating quantitative analysis via SmartPLS and IBM SPSS, to examine the interconnections among a multitude of variables, including corporate performance, Lean practices, top management commitment, and Industry 4.0 adoption. The implementation of rigorous methods in this study contributes to the overall credibility and dependability of the results. Aspects of Implementation Difficulties: The study's unanticipated discoveries, including the absence of immediate enhancements in corporate performance following the adoption of Lean 4.0, stimulate reflection on the difficulties associated with implementation and the gradual adjustment that organisations must make to this integrated methodology. This knowledge regarding prospective obstacles is beneficial for organisations undertaking comparable transformation endeavours. In its entirety, this research endeavour elevates the discipline by furnishing empirical data and theoretical elucidation about the implementation of Lean 4.0 methodologies within the pharmaceutical sector of Ghana. The integration of Lean principles and Industry 4.0 technologies presents practical implications for pharmaceutical companies aiming to optimise their manufacturing processes, enhance quality, and drive performance. Additionally, it underscores the criticality of leadership commitment in this pursuit.

## 7 Conclusion

This study aimed to establish a comprehensive top management commitment to implement Lean 4.0 within the pharmaceutical sector of Ghana—an industry critical for the nation's healthcare system and economic progress. The research addressed the Success Factors of Top management commitment and Implementation of Lean 4.0 adoption, identified implementation challenges, and delved into the impact of top management commitment on driving this transformative change. Employing an explanatory research design and survey methods rooted in a positivist ideology, the study collected data from personnel in Ghanaian pharmaceutical companies. Structural equation modelling was applied for analysis.

The findings underscore the pivotal role of strong top management commitment, indicating a substantial and positive influence on both lean methodology implementation and Industry 4.0 technology adoption. This highlights the critical importance of leadership dedication in steering strategic initiatives like Lean 4.0. Additionally, the study revealed that the adoption of lean practices and Industry 4.0 technologies significantly contributes to Lean 4.0 implementation, notwithstanding requiring meticulous management.

However, the research noted that Lean 4.0 did not exhibit an immediate substantial positive impact on corporate performance, suggesting that its benefits may manifest over time. Conversely, the individual implementation of lean principles and Industry 4.0 technology demonstrated positive effects on business performance.

In summary, this work makes several noteworthy contributions. It underscores the vital role of executive-level commitment in facilitating Lean 4.0 adoption. The study advocates for a systematic integration of Lean and Industry 4.0, coupled with effective change management, to realize the promised efficiency and innovation enhancements. Ultimately, this research aims to catalyse the acquisition of new knowledge and stimulate dialogue, fostering the advancement of the Ghanaian pharmaceutical industry toward a more efficient and technologically advanced future.

### 7.1 Limitations and future directions

Although this study provides vital insights into the application of Lean 4.0 in the pharmaceutical industry of Ghana, it is crucial to recognise its limits and highlight areas for further research. An inherent constraint of the research is its exclusive concentration on the pharmaceutical industry of Ghana, hence restricting the applicability of the results to other sectors or geographical areas. Subsequent studies may investigate the suitability of the suggested framework in other industries or nations, taking into account the distinctive contextual elements that could impact the adoption of Lean 4.0. Another constraint is the dependence on survey methodologies and self-reported data, which might potentially create biases or inaccuracies in measurement. To get a more thorough knowledge of the problems and variables that contribute to the successful adoption of Lean 4.0, future studies should enhance survey data by using qualitative interviews or observational research. Furthermore, while the research emphasizes the significance of top management dedication in advancing Lean 4.0 projects, it does not extensively explore the precise processes by which this dedication impacts the results of implementation. Further research might examine the impact of different leadership styles, organisational culture, and communication tactics on the facilitation or obstruction of Lean 4.0 implementation. Moreover, the study's discovery that Lean 4.0 did not result in significant immediate benefits for corporate performance indicates the need for longitudinal research to evaluate the lasting consequences of Lean 4.0 projects. Subsequent research might monitor performance indicators over some time to ascertain the long-term viability and expandability of Lean 4.0 methodologies in enhancing organisational results. Although this study offers useful insights into the application of Lean 4.0 in the pharmaceutical industry of Ghana, there are still various areas that future research might investigate. Researchers may enhance their knowledge of Lean 4.0 adoption and its impact on organisational performance and competitiveness by overcoming these constraints and expanding on the results of this study.

## CRediT authorship contribution statement

**Michelle Grace Tetteh:** Writing – review & editing, Writing – original draft, Validation, Methodology, Investigation, Formal analysis, Data curation, Conceptualization. **Sumit Gupta:** Writing – review & editing, Validation, Methodology, Investigation, Formal analysis. **Mukesh Kumar:** Writing – review & editing, Writing – original draft, Validation, Methodology, Investigation, Formal analysis. **Hana Trollman:** Writing – review & editing, Visualization, Validation, Supervision, Methodology. **Konstantinos Salonitis:** Writing – review & editing, Supervision, Resources, Project administration, Funding acquisition. **Sandeep Jagtap:** Writing – review & editing, Validation, Supervision, Resources, Project administration, Funding acquisition, Conceptualization.

## Declaration of competing interest

The authors declare that they have no known competing financial interests or personal relationships that could have appeared to influence the work reported in this paper.

## Footnotes

Appendix A Supplementary data to this article can be found online at https://doi.org/10.1016/j.heliyon.2024.e36677.
