# Supplementary material for: Pharma 4.0: A deep dive top management commitment to successful Lean 4.0 implementation in Ghanaian pharma manufacturing sector
Source: Heliyon. 2024 Aug 29;10(17):e36677. doi: 10.1016/j.heliyon.2024.e36677 (PMC11408067; doi:10.1016/j.heliyon.2024.e36677)
Supplement: Multimedia component 1 [file mmc1.docx]

**Appendix A**

Ghanaian Pharma Survey

Q 1. Level of job position

- Executive or senior management (1)
- Middle management (2)
- First-level management (3)
- Intermediate or experienced (Senior staff) (4)
- Entry–level (5)

Q 2. What is your area of work?

- R&D/Product Design & Development (1)
- Purchasing/Supply Chain (2)
- Production/Manufacturing (3)
- Marketing & Sales (4)
- Information Technology (5)
- Human Resource Management (6)
- Finance (7)
- Other (8) __________________________________________________

Q 3. Size of your organization.

- Small (Less than 50 employees) (1)
- Medium (Between 50 to 250 employees) (2)
- Large (More than 250 employees) (3)

Q 4. How do you rate the level of top management commitment with respect to the following in your business? (1-Not at all, 2–Low, 3–Slightly, 4– Neutral, 5–Moderately, 6–Very, 7–Extremely)

|  | 1 | 2 | 3 | 4 | 4 | 5 | 6 | 7 |
| --- | --- | --- | --- | --- | --- | --- | --- | --- |

| Act as a change leader () | 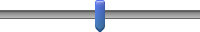 |
| --- | --- |
| Inclination towards better quality and performance () | 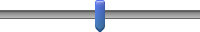 |
| Cross-functional cooperation and integration at all levels of the company () | 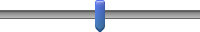 |
| Willingness towards the adoption of Sustainable and Smart Manufacturing () | 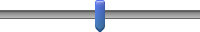 |
| Emphasis on environmental management and improving eco-efficiency () | 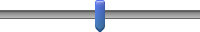 |

Q 5. How do you think the Lean Practices can be applied to improve the pharma practice in drug manufacturing in Ghana? (1-Not at all, 2–Low, 3–Slightly, 4– Neutral, 5–Moderately, 6–Very, 7–Extremely)

|  | 1 | 2 | 3 | 4 | 4 | 5 | 6 | 7 |
| --- | --- | --- | --- | --- | --- | --- | --- | --- |

| Continuous improvement () | 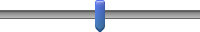 |
| --- | --- |
| Value Stream Mapping (VSM) () | 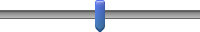 |
| Management techniques ( Poka-Yoke, Single Minute Exchange of Dies (SMED) and Heijunka (Production Levelling). () | 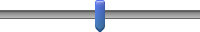 |
| Encouraging employee () | 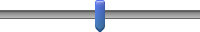 |
| Total Productive Maintenance (TPM) () | 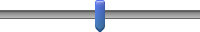 |
| Investing in training and development () | 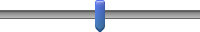 |

Q 6. How do you think Industry 4.0 can be used to improve the pharma practice in drug manufacturing in Ghana? (1-Not at all, 2–Low, 3–Slightly, 4– Neutral, 5–Moderately, 6–Very, 7–Extremely)

|  | 1 | 2 | 3 | 4 | 4 | 5 | 6 | 7 |
| --- | --- | --- | --- | --- | --- | --- | --- | --- |

| Smart manufacturing technologies for real-time by collecting and analysing data () | 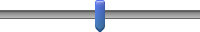 |
| --- | --- |
| Artificial intelligence and machine learning to identify patterns of personalised effective drugs () | 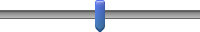 |
| Utilizing the Internet of Things (IoT) to track and monitor the supply chain to optimal conditions to maintain quality and safety () | 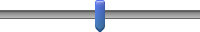 |
| Virtual reality and augmented reality tools for training, to improve safety and accuracy in drug manufacturing processes () | 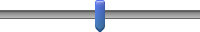 |
| Adoption of Blockchain Technology () | 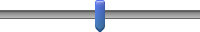 |

Q 7. Lean 4.0 represents the next evolution of the Lean management philosophy, incorporating digital technology and Industry 4.0 principles. (1-Not at all, 2–Low, 3–Slightly, 4– Neutral, 5–Moderately, 6–Very, 7–Extremely)

|  | 1 | 2 | 3 | 4 | 4 | 5 | 6 | 7 |
| --- | --- | --- | --- | --- | --- | --- | --- | --- |

| Leadership commitment and support () | 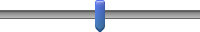 |
| --- | --- |
| Employee engagement and participation () | 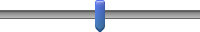 |
| Adequate training and skill acquisition () | 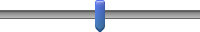 |
| Availability of technology and infrastructure () | 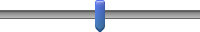 |
| Stakeholder involvement () | 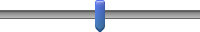 |
| Continuous monitoring and evaluation () | 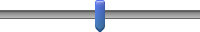 |

Q 8. How does adopting Lean 4.0 contributes to overall corporate performance? (1-Not at all, 2–Low, 3–Slightly, 4– Neutral, 5–Moderately, 6–Very, 7–Extremely)

|  | 1 | 2 | 3 | 4 | 4 | 5 | 6 | 7 |
| --- | --- | --- | --- | --- | --- | --- | --- | --- |

| Stakeholder engagement: (such as customers, employees, suppliers) () | 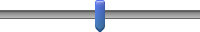 |
| --- | --- |
| Ethical business practices: (hazardous chemicals, integrity, and fairness) () | 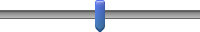 |
| Human rights: (individuals rights are not violated in the production process) () | 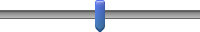 |
| Cost-effectiveness: (sustainability principles) () | 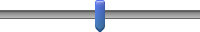 |
| Resource efficiency: (energy, water, and materials) () | 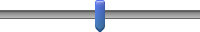 |
| Financial viability () | 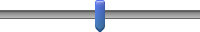 |
| Stakeholder value: (shareholders, employees, and local communities) () | 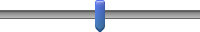 |
| The company’s financial viability is aligned with sustainability principles and practices () | 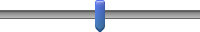 |

**Appendix B**

**B1. Survey data analysis**

Table B1 illustrates the overall mean score for Top Management Commitment (TMC), which stands at 6.17 out of 7. This high mean score suggests a substantial agreement among respondents regarding top management's commitment to sustainability and smart manufacturing. The relatively low standard deviation of 0.76 indicates limited variability in the responses, highlighting a consistent trend. Examining individual items, mean scores range from 6.1 to 6.23, further affirming consistently high agreement levels. The standard deviation of 0.76 reinforces the minimal variation in the replies.

Within the individual items, TMC_2 exhibits the lowest average, indicating a relatively lower consensus on management's inclination towards quality and performance. Conversely, TMC_4 secures the highest rank in terms of the organization's eagerness to embrace sustainable/smart manufacturing, with an average score of 6.1, signifying a high level of consensus among respondents.

**Table B1.** Descriptive Results on Top Management Commitment

| Code | Top Management Commitment | Min | Max | Mean | SD |
| --- | --- | --- | --- | --- | --- |
| TMC_1 | Act as a change leader | 2 | 7 | 6.11 | 0.881 |
| TMC_2 | Inclination towards better quality and performance | 1 | 7 | 6.1 | 0.932 |
| TMC_3 | Cross-functional cooperation and integration at all levels of the company | 1 | 7 | 6.22 | 0.945 |
| TMC_4 | Willingness towards the adoption of Sustainable and Smart Manufacturing | 1 | 7 | 6.23 | 1.006 |
| TMC_5 | Emphasis on environmental management and improving eco-efficiency | 1 | 7 | 6.21 | 0.949 |
| TMC | Composite Score | 2.4 | 7 | 6.1735 | 0.76082 |

Table B2 highlights that the overall Lean Practices (LP) mean is 6.15 out of 7, suggesting a broad consensus among respondents that their firms have embraced various lean practices. The standard deviation of 0.78 indicates minimal dispersion in the responses, underscoring the uniformity in the agreement. Analyzing individual items, mean scores range from 6.05 to 6.31, illustrating consistently high scores across specific practices. These findings imply the pervasive implementation of lean initiatives, encompassing practices such as continuous improvement, value stream mapping, Total Productive Maintenance (TPM), and investment in training.

**Table B2** Descriptive Results on Lean Practices

| Code | Lean Practices | Min | Max | Mean | SD |
| --- | --- | --- | --- | --- | --- |
| LP_1 | Continuous improvement | 2 | 7 | 6.06 | 0.867 |
| LP_2 | Value Stream Mapping (VSM) | 1 | 7 | 6.08 | 1.021 |
| LP_3 | Management techniques ( Poka-Yoke, Single Minute Exchange of Dies (SMED) and Heijunka (Production Levelling). | 1 | 7 | 6.05 | 1.199 |
| LP_4 | Encouraging employee | 1 | 7 | 6.19 | 0.87 |
| LP_5 | Total Productive Maintenance (TPM) | 1 | 7 | 6.31 | 0.951 |
| LP_6 | Investing in training and development | 1 | 7 | 6.2 | 0.929 |
| LP | Composite Score | 2 | 7 | 6.1492 | 0.77867 |

The overall mean from Table B3 reveals that Lean 4.0 is scored at 6.27 out of 7, indicating a substantial agreement among respondents that their firms have successfully transitioned lean practices towards Industry 4.0. The minimal standard deviation of 0.70 underscores the consistency and limited variability in the responses. Examining specific items, mean scores range from 6.17 to 6.38, affirming consistently high ratings across the evaluated aspects. These findings suggest that companies have effectively integrated lean methodologies with Industry 4.0 technologies, incorporating training initiatives and fostering stakeholder involvement in the process.

**Table B3** Descriptive Results on Lean 4.0

| Code | Lean 4.0 | Min | Max | Mean | SD |
| --- | --- | --- | --- | --- | --- |
| L4.0_1 | Leadership commitment and support | 3 | 7 | 6.22 | 0.784 |
| L4.0_2 | Employee engagement and participation | 1 | 7 | 6.17 | 1.032 |
| L4.0_3 | Adequate training and skill acquisition | 1 | 7 | 6.25 | 1.091 |
| L4.0_4 | Availability of technology and infrastructure | 1 | 7 | 6.29 | 0.953 |
| L4.0_5 | Stakeholder involvement | 3 | 7 | 6.38 | 0.832 |
| L4.0_6 | Continuous monitoring and evaluation | 3 | 7 | 6.33 | 0.715 |
| L4.0 | Composite Score | 2.83 | 7 | 6.2735 | 0.69873 |

Table B4 exhibits a minimal standard deviation of 0.74, indicating limited dispersion in the responses. When considering specific items, the mean scores range from 6.02 to 6.27, consistently reflecting high ratings across the board. Respondents overwhelmingly agree that their organizations have effectively incorporated a variety of Industry 4.0 technologies. The lowest average is observed for I4.0_2, specifically for artificial intelligence/machine learning applications, while the highest average is recorded for I4.0_4, highlighting virtual/augmented reality tools. Despite slight variations, all averages affirm a consensus that Industry 4.0 technologies are actively being implemented. These findings suggest that companies are harnessing technologies such as the Internet of Things (IoT), blockchain, artificial intelligence (AI), and virtual reality to empower smart manufacturing capabilities.

**Table B4.** Descriptive Results on Industry 4.0 Technology

| Code | Industry 4.0 Technology | Min | Max | Mean | SD |
| --- | --- | --- | --- | --- | --- |
| I4.0_1 | smart manufacturing technologies for real-time by collecting and analysing data | 1 | 7 | 6.13 | 0.955 |
| I4.0_2 | Artificial intelligence and machine learning to identify patterns of personalised effective drugs | 1 | 7 | 6.02 | 1.178 |
| I4.0_3 | Utilizing the Internet of Things (IoT) to track and monitor the supply chain to optimal conditions to maintain quality and safety | 1 | 7 | 6.17 | 0.98 |
| I4.0_4 | virtual reality and augmented reality tools for training, to improve safety and accuracy in drug manufacturing processes | 1 | 7 | 6.27 | 0.924 |
| I4.0_5 | Adoption of Blockchain technology | 2 | 7 | 6.2 | 0.945 |
| I4.0 | Composite Score | 2.8 | 7 | 6.1569 | 0.74006 |

The insights derived from Table B5 suggest that companies are attaining robust triple-bottom-line performance, encompassing economic, social, and environmental parameters. The mean scores range from 6.15 to 6.36, consistently reflecting high levels of agreement. The lowest mean is observed for CP_3, specifically regarding human rights protection, while the highest mean is noted for CP_4, emphasizing cost-effectiveness. Despite slight variations, all mean scores indicate a shared consensus that corporate performance is robust across sustainability dimensions.

The descriptive data further reveal that pharmaceutical workers possess a strong perception of the company's sustainable policies, underscoring a solid corporate performance. The elevated mean averages and reduced standard deviations reinforce this positive assessment. Overall, the data strongly suggest that firms are effectively achieving robust triple-bottom-line performance concerning economic, social, and environmental aspects.

**Table B5** Descriptive Results on Corporate Performance

| Code | Corporate Performance | Min | Max | Mean | SD |
| --- | --- | --- | --- | --- | --- |
| CP_1 | Stakeholder engagement: (such as customers, employees, suppliers,). | 1 | 7 | 6.2 | 0.859 |
| CP_2 | Ethical business practices: (hazardous chemicals, integrity, and fairness). | 2 | 7 | 6.23 | 0.844 |
| CP_3 | Human rights: (individuals rights are not violated in the production process) | 1 | 7 | 6.15 | 1.323 |
| CP_4 | Cost-effectiveness: (sustainability principles). | 1 | 7 | 6.36 | 0.966 |
| CP_5 | Resource efficiency: (energy, water, and materials) | 1 | 7 | 6.3 | 1.05 |
| CP_6 | Financial viability | 1 | 7 | 6.26 | 1.176 |
| CP_7 | Stakeholder value: (shareholders, employees, and local communities. | 1 | 7 | 6.31 | 0.86 |
| CP_8 | The company’s financial viability is aligned with sustainability principles and practices | 3 | 7 | 6.33 | 0.788 |
| CP | Composite Score | 3 | 7 | 6.2686 | 0.69466 |

**Table B6.** Hypotheses Table

| **Hypothesis** | **Path** | **T-value** | **Coefficient (P-value)** | **Conclusion** |
| --- | --- | --- | --- | --- |
| **H_1_** | TMC 🡪 LP | 17.47 | 0.82; p < 0.01 | Supported |
| **H_2_** | TMC 🡪 I4.0 | 21.78 | 0.78; p < 0.01 | Supported |
| **H_3a_** | LP 🡪 L4.0 | 2.11 | -0.09; p < 0.05 | Not Supported |
| **H3b** | L4.0 🡪 CP | 1.70 | 0.17; P > 0.05 | Not Supported |
| **H3c** | I4.0 🡪 L4.0 | 4.04 | 0.52; p < .01 | Supported |
| **H4** | LP 🡪 CP | 3.51 | 0.27; p < .01 | Supported |
| **H5** | I4.0 🡪 CP | 2.15 | 0.20; p < .05 | Supported |
